# Relationship between early venous filling after thrombectomy and intracranial hemorrhage and prognosis in patients with acute ischemic stroke: a systematic review and meta-analysis

**DOI:** 10.3389/fneur.2025.1647906

**Published:** 2026-01-14

**Authors:** Xiaoyu Wu, Chao Li, Mingchao Shi, Kangjia Song, Mingchen Zhang, Siyuan Wang, Shouchun Wang

**Affiliations:** Stroke Center, Department of Neurology, The First Hospital of Jilin University, Changchun, China

**Keywords:** acute ischemic stroke, early venous filling, endovascular treatment, prognosic, thrombectomy

## Abstract

**Objective:**

This study aims to assess the relationship between early venous filling (EVF) observed during mechanical thrombectomy and 90-day functional independence (mRS ≤ 2) as well as postoperative intracranial hemorrhage (ICH) and symptomatic intracranial hemorrhage (SICH), in patients with acute ischemic stroke.

**Methods:**

We conducted a systematic search of the PubMed, EMBASE, Web of Science, and Cochrane Library databases up to September 26, 2024, following the PRISMA 2020 guidelines. The primary clinical outcomes were defined as 90-day functional independence (mRS ≤ 2) and SICH, while the secondary clinical outcome was ICH. Odds ratios (OR) and 95% confidence intervals (CI) were estimated using a random-effects model.

**Results:**

A total of six studies that met the inclusion criteria were analyzed. Meta-analysis demonstrated that patients with EVF had a significantly reduced likelihood of achieving 90-day functional independence compared to patients without EVF (OR 0.42, 95% CI 0.18–0.99, *P* = 0.05). Moreover, patients with EVF exhibited a markedly higher risk of post-procedural SICH than patients without EVF (OR 4.68, 95% CI 2.20–9.96, *P* < 0.001). Additionally, EVF was strongly linked to an increased incidence of post-procedural ICH (OR 5.73, 95% CI 2.57–12.82, *P* < 0.0001).

**Conclusion:**

EVF may serve as an intraoperative angiographic marker associated with a higher incidence of postoperative ICH, SICH, and poorer 90-day functional independence.

**Systematic review registration:**

https://www.crd.york.ac.uk/PROSPERO/, identifier CRD42024595410.

## Introduction

Stroke ranks as the second leading cause of disability and death globally, creating a high disease and economic burden, particularly in low- and middle-income countries. Approximately 87% of stroke cases are ischemic (1). Therefore, prompt and appropriate treatment in acute ischemic stroke patients is essential to reduce mortality and morbidity (2).

Mechanical thrombectomy is the established standard for treating acute ischemic stroke (3). Compared to pharmacological treatment alone, mechanical thrombectomy can significantly enhance patient prognosis within 6–24 h of stroke onset (4) or even more than 24 h (5). However, nearly half of the patients do not have a good prognosis after mechanical thrombectomy (6), which is usually due to complications such as symptomatic intracranial hemorrhage (SICH), ineffective recanalization, or vascular entrapment (7). Early venous filling (EVF) observed during mechanical thrombectomy may be associated with the conversion to intracranial hemorrhage (ICH).

EVF is a relatively common phenomenon in mechanical thrombectomy that usually manifests itself under digital subtraction angiography (DSA) assessment as early visualization of the venous system during the arterial or capillary phase. The mechanism of EVF is not well-understood. However, it is known that ischemia and hypoxia in brain tissue can disrupt cellular regulation or cause cell death, which further results in capillary dilatation or disruption of capillary integrity (8). EVF may be associated with postoperative ICH, especially symptomatic intracranial hemorrhage (SICH), but its association with clinical prognosis remains unclear.

Given the conflicting results and limited sample sizes of existing single-center studies, a systematic review and meta-analysis are necessary to provide a more robust estimate of the association between EVF and clinical outcomes. This study aims to assess the relationship between EVF during mechanical thrombectomy, 90-day functional independence, and postoperative ICH in patients with acute ischemic stroke, which will provide a reference for the perioperative treatment of patients.

## Methods

This systematic review and meta-analysis followed the Preferred Reporting Items for Systematic Reviews and Meta-Analyses report (9) and was registered in PROSPERO (CRD42024595410).

### Search strategy

A systematic search was conducted in PubMed, EMBASE, Web of Science, and the Cochrane Library up to 26 September 2024. Search terms included the following keywords or MeSH subject terms: Ischemic stroke, Acute Ischemic Stroke, Cryptogenic Ischemic Stroke, Cryptogenic Embolism Stroke, early, venous filling, luxury perfusion, etc. To ensure the comprehensiveness of the retrieved literature, we manually checked the reference lists of relevant studies (Supplementary Table 1).

### Inclusion and exclusion criteria

Studies were included if they met the following conditions: (1) Participants were diagnosed with acute ischemic stroke, (2) The patients underwent mechanical thrombectomy, (3) The operator performed an intraoperative assessment of EVF by digital subtraction angiography (DSA), (4) The study endpoints included ICH or SICH, as shown by imaging, and (5) The study design was either a prospective or retrospective cohort.

Studies were excluded if they met any of the following: (1) Conferences, reviews, letters, commentaries, case reports, or animal studies; (2) Non-English language literature; (3) Studies for which relevant data were not available; and (4) Studies with inconsistent outcome indicators.

### Data extraction

Two experienced researchers (XYW and MCZ) independently extracted relevant data and cross-checked them. Disagreements were evaluated and resolved by a third researcher (KJS). The extracted data included: basic information about the study (first author, corresponding author, and publication year), baseline characteristics of the patients (age, sex, hypertension, diabetes mellitus, atrial fibrillation, anticoagulant/antiplatelet medication use, site of vascular occlusion, etc.), rate of premature venous display, rate of postoperative bleeding (both ICH and SICH), and 90-day functional independence rate [modified Rankin Scale (mRS) ≤ 2].

### Quality assessment

The Newcastle-Ottawa Scale (NOS) was used to evaluate all cohort studies included in this analysis, which is scored in terms of (1) assessment of the selection of exposed and unexposed cohorts, (2) between-group comparability of the two cohorts, and (3) assessment of outcomes (10). This assessment was also independently conducted and cross-checked by two experienced researchers (XYW and MCZ), with disagreements tested and resolved by a third researcher (KJS). A final score of ≥7 was regarded as indicating a high-quality study.

### Statistical analyses

We used RevMan 5.4 software (Review Manager, version 5.4, Cochrane Collaboration, London, UK) to create forest plots and Stata 15 (StataCorp LLC, College Station, TX, USA) for heterogeneity tests and sensitivity analyses. We calculated the odds ratio (OR) of early venous presentation to postoperative ICH and functional independence and their 95% confidence intervals (CI) using the data that had been extracted and pooled from each study. Heterogeneity was assessed with the *I*^2^ statistic; significant heterogeneity was indicated when *P* < 0.10 and *I*^2^ > 50%. In the presence of heterogeneity, a random-effects model was used for combined analyses; whereas a fixed-effects model was used when heterogeneity was absent. We also produced funnel, labeled, and star plots to assess publication bias and sources of heterogeneity in the included studies. At the same time, we performed sensitivity analyses using RevMan 5.4 software to sequentially exclude individual studies to evaluate their impact on the pooled results.

## Results

### Search results

**Figure 1:**
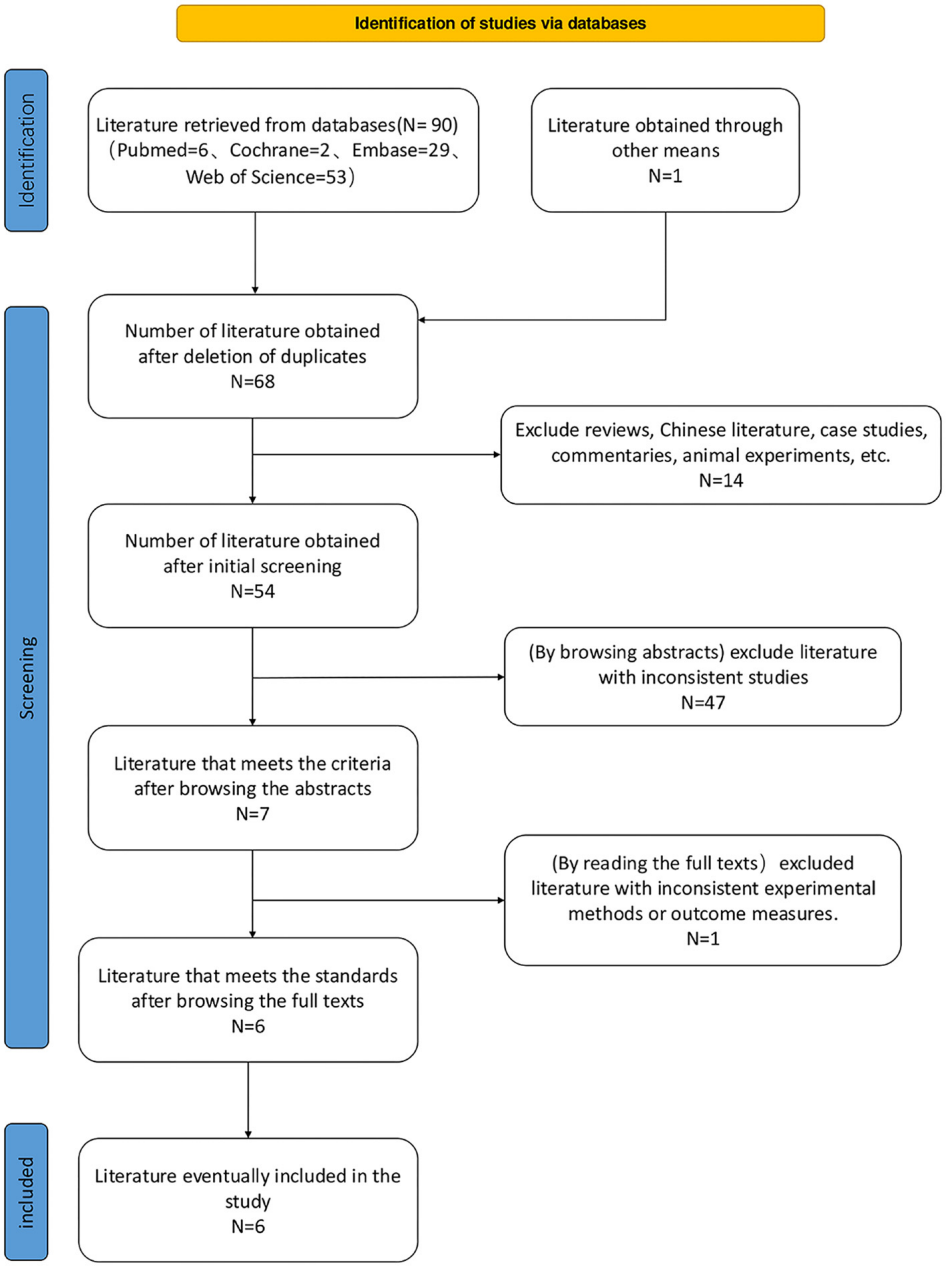
Flowchart of literature search literature screening and literature inclusion.

We preliminarily obtained 91 relevant papers through a refined search strategy and a case-by-case verification of the retrieved citations, of which 6, 29, 53, and 2 were retrieved from PubMed, EMBASE, Web of Science, and Cochrane Library databases, respectively. One was obtained from literature citations. After removing duplicates, 68 articles remained. Following a review of abstracts and titles, we excluded irrelevant literature, reviews, Chinese literature, case reports, and conferences, leaving seven articles. Following a full-text review, one study was excluded owing to inconsistent outcome indicators, resulting in a final selection of six original studies (8, 11–15) (Figure 1).

### Characteristics and quality assessment of included studies

The six original studies (Table 1) had a total of 1,101 patients, of whom 292 (26.5%) presented with EVF. Two of the original studies extracted detailed baseline data (age, sex, hypertension, diabetes mellitus, and atrial fibrillation) related to the group presenting with EVF vs. the group without EVF. Analysis of these baseline data revealed a significant difference in smoking between the two groups (Supplementary Table 2). However, the remaining four original studies did not yield data on relevant baseline characteristics; thus, the results of the analyses derived from these studies may be limited in terms of general applicability. In addition, four studies reported 90-day functional independence (mRS ≤ 2), five reported the incidence of SICH, and four reported data on postoperative ICH rates. The six papers included in this study scored 7–9 on the NOS when assessed; therefore, all six are considered high-quality studies (Supplementary Table 3).

**Table 1:** Basic information on the inclusion of literature.

**References**	**Research methods**	**EVF confirmation method**	**EVF**	**N-EVF**	**Total**
Li et al. (12)	Retrospective cohort study	DSA	45	304	349
Cartmell et al. (8)	Retrospective cohort study	DSA	15	49	64
Janvier et al. (13)	Retrospective cohort study	DSA	146	256	402
Elands et al. (14)	Retrospective cohort study	DSA	33	114	147
Shimonaga et al. (15)	Retrospective cohort study	DSA	22	13	35
Ohta et al. (11)	Retrospective cohort study	DSA	31	73	104

EVF, early venous filling; N-EVF, non-early venous filling; DSA, digital subtraction angiography.

#### Primary clinical outcomes: 90-day functional independence (mRS ≤ 2) and SICH

**Figure 2:**
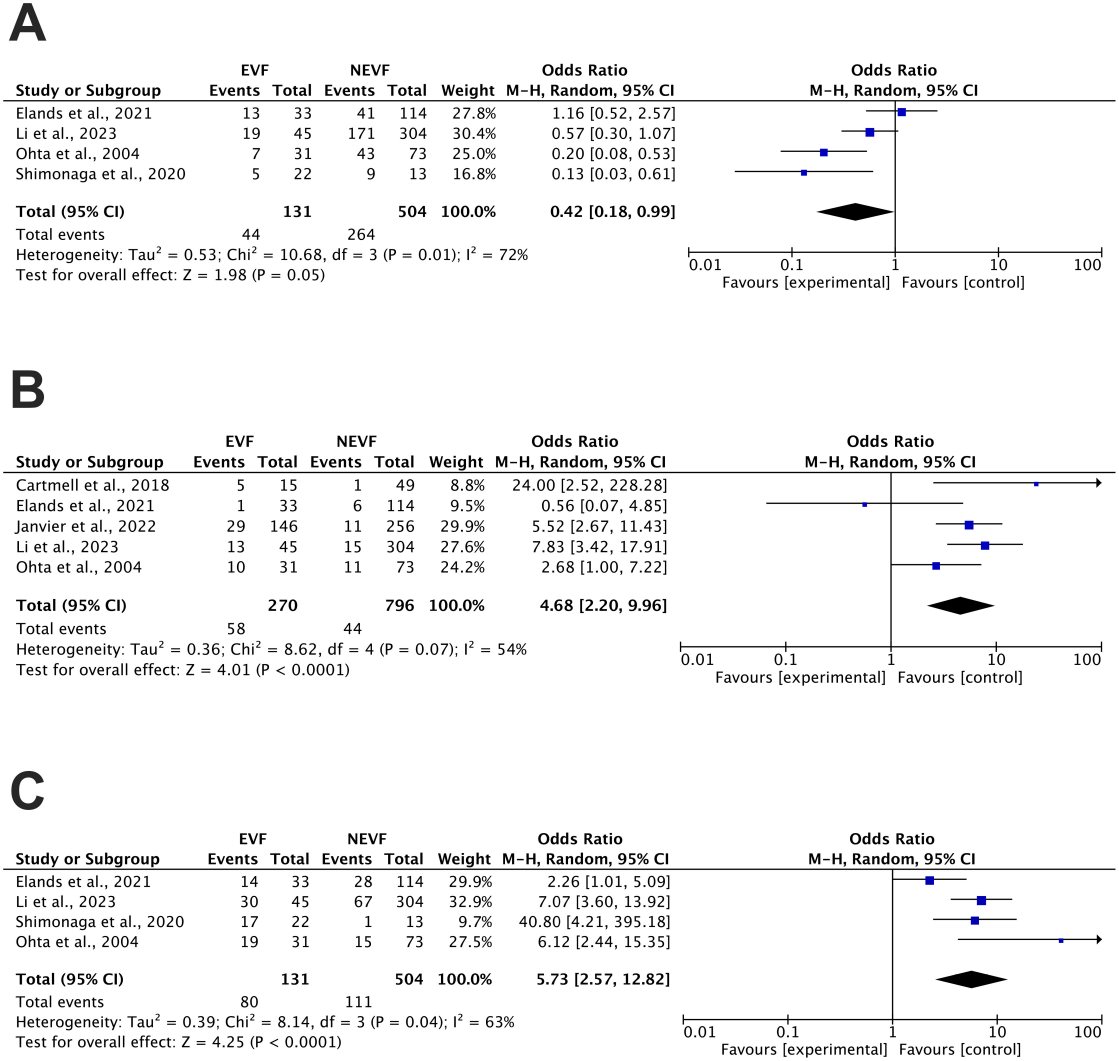
Forest plot of outcomes comparing the presence of EVF with the absence of EVF. **(A)** 90-day functional independence (mRS ≤ 2), **(B)** Symptomatic intracranial hemorrhage (SICH), and **(C)** Intracranial hemorrhage (ICH). EVF, early venous filling; N-EVF, non-early venous filling; CI, confidence interval.

The analysis of the 90-day functional independence included four studies, which showed a significant difference between patients with and without EVF after mechanical thrombectomy (33.6 vs. 52.4%, OR 0.42, 95% CI 0.18–0.99, *P* = 0.05). Heterogeneity analysis indicated substantial heterogeneity (*I*^2^ = 72% and *P* = 0.01); therefore, a random-effects model was applied (Figure 2A).

For SICH outcomes, five studies were analyzed, showing a significantly higher incidence of SICH in patients with EVF compared to those without EVF (21.5 vs. 5.5%, OR 4.68, 95% CI 2.20–9.96, *P* < 0.001). Heterogeneity analysis indicated moderate heterogeneity (*I*^2^ = 54% and *P* = 0.07); therefore, a random-effects model was applied (Figure 2B).

#### Secondary clinical outcome: ICH

The analysis of ICH outcome included four studies, demonstrating a significantly higher incidence of ICH in patients with EVF compared to those without EVF (61.1 vs. 22.0%, OR 5.73, 95% CI 2.57–12.82, *P* < 0.0001). Heterogeneity analysis indicated moderate heterogeneity (*I*^2^ = 63% and *P* = 0.04); therefore, a random-effects model was applied (Figure 2C).

### Sensitivity analysis and publication bias test

**Figure 3:**
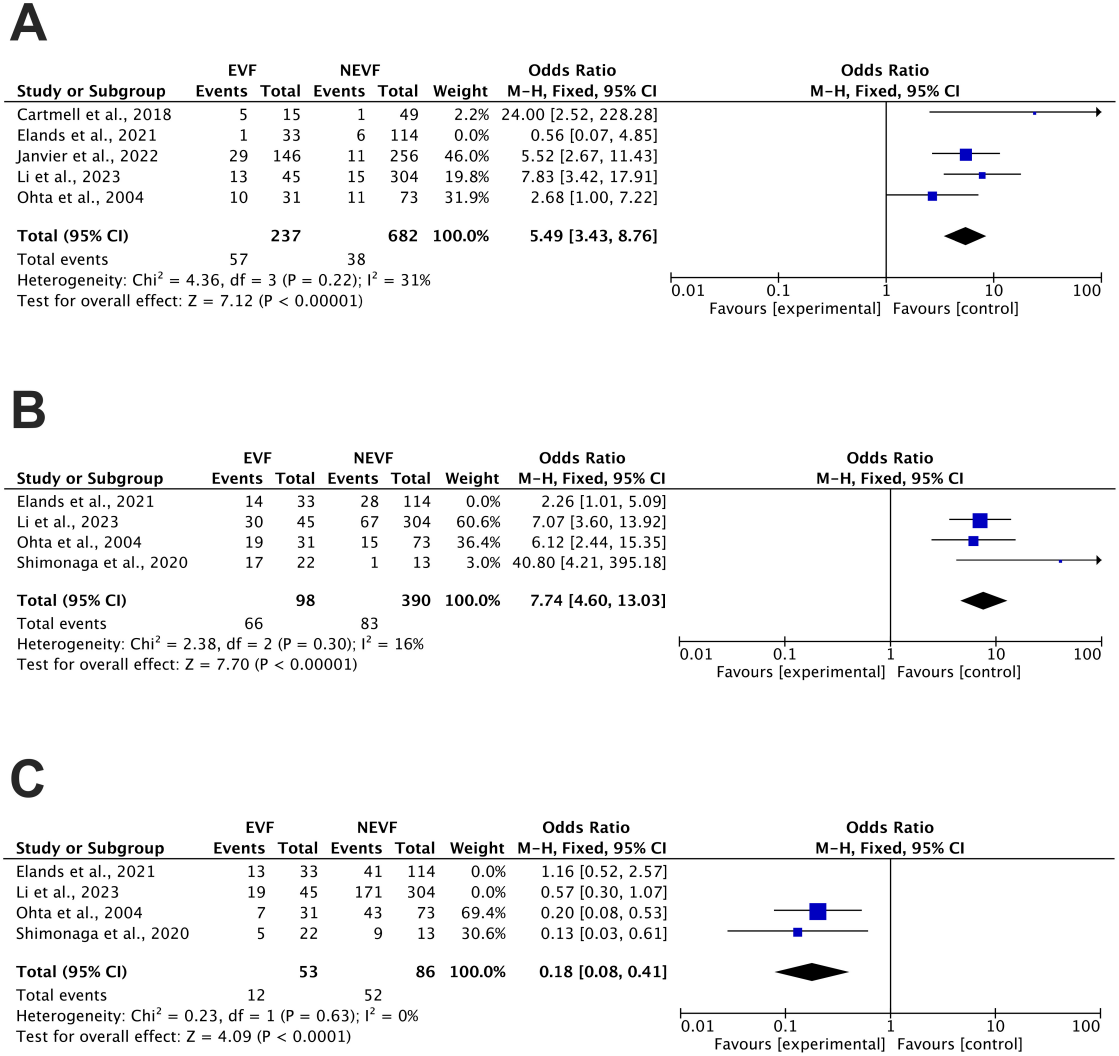
Adjusted forest plot of outcomes comparing the presence of EVF with the absence of EVF **(A)** Symptomatic intracranial hemorrhage (SICH), **(B)** Intracranial hemorrhage (ICH), and **(C)** 90-day functional independence (mRS ≤ 2). EVF, early venous filling; N-EVF, non-early venous filling; CI, confidence interval.

Given the heterogeneity observed in the clinical outcome analyses, sensitivity analyses and publication bias tests were conducted for each outcome. We derived adjusted results, revealing a significant difference in the incidence of SICH in patients between those with EVF and those without it after mechanical thrombectomy (24.1 vs. 5.6%, OR 5.49, 95% CI 3.43–8.76, *P* < 0.00001). Heterogeneity analysis showed *I*^2^ = 31% and *P* = 0.22 (Figure 3A). An adjusted funnel plot was generated, with all study sites showing a symmetrical distribution trend (Supplementary Figure 1A). In summary, the adjusted results demonstrate high robustness. Using the same methodology, the analysis and adjustment of the rate of ICH for the secondary outcome revealed a significant difference in incidence between patients with EVF and those without it (67.3 vs. 21.3%, OR 7.74, 95% CI 4.60–13.03, *P* < 0.00001). Heterogeneity analysis indicated low heterogeneity (*I*^2^ = 16% and *P* = 0.30; Figure 3B). Combined with the funnel plot, this demonstrated the high robustness of the secondary outcome after adjustment (Supplementary Figure 1B). Similarly, we evaluated the primary functional outcome of 90-day functional independence. After removing the two influential studies identified in the initial pooled analysis, the remaining studies demonstrated a stable association between EVF and a lower likelihood of achieving 90-day functional independence (22.6 vs. 60.5%; OR 0.18, 95% CI 0.08–0.41; *P* < 0.0001). Heterogeneity analysis revealed no detectable heterogeneity (*I*^2^ = 0% and *P* = 0.63; Figure 3C). As only two studies were included in this sensitivity analysis, these findings should be interpreted cautiously. Nevertheless, the direction and magnitude of the association were highly consistent with the trends observed in the primary analysis, indicating that the overall relationship is unlikely to be driven by outlier effects.

## Discussion

This meta-analysis identified a significant association between EVF and 90-day functional independence after mechanical thrombectomy. However, caution is warranted, as differences in patient composition, EVF evaluation, and peri-operative practice patterns across cohorts may have influenced the observed associations. In this context, EVF is more appropriately regarded as an intraoperative angiographic marker associated with adverse outcomes, rather than a proven independent predictor of prognosis. Previous research has shown that ICH is one of the most serious complications in ischemic stroke treatment (16), and SICH, resulting from significant cerebral edema and mass effects, may be associated with worse 90-day functional outcomes (17, 18). Therefore, we speculate that the poor prognosis observed in patients with EVF may be linked to a higher likelihood of ICH or SICH, which could lead to further deterioration of the patient's neurological function. In addition, EVF is usually more readily observed at the level of the basal ganglia, where the vascular distribution is terminal in nature and prone to disruption of the normal arteriovenous return structure due to ischemia. This area is functionally important, and such infarctions may also ultimately result in worse clinical outcomes and a higher mortality rate.

The physiological mechanism of EVF may be related to the overexpression of nitric oxide synthase in brain tissue after ischemia. The overexpression of endothelial nitric oxide synthase increases nitric oxide production (19), which has a potent vasodilatory effect and simultaneously exacerbates vascular endothelial damage (20). This, in turn, increases blood flow to the region of the damaged brain tissue, potentially leading to rapid venous phase blood arrival, manifesting as EVF. As ischemic metabolism cascades, this impairment of cerebral autoregulation accelerates the breakdown of the blood-brain barrier, which may subsequently result in hemorrhagic transformation or reperfusion hemorrhage (20, 21).

EVF may be associated with intracranial hyperperfusion after mechanical thrombectomy (15). From this perspective, EVF may manifest as an intraoperative angiographic sign linked to complications, such as postoperative ICH, SICH, and malignant cerebral edema (12). However, the relationship between EVF and the 90-day functional independence remains controversial.

Individual studies have reported inconsistent associations between EVF and 90-day functional independence. Li et al. reported no significant association between EVF and 90-day functional independence, though a clinical trend toward a worse prognosis in the EVF group (42.2 vs. 56.3%; OR 0.568, *P* = 0.08) (12). With only 45/349 patients (12.9%) exhibiting EVF, the reliability of the final results may be limited owing to insufficient subgroup and overall sample sizes. In contrast, Elands et al. observed a non-significant trend in the opposite direction (52.6 vs. 40.7%, *P* > 0.05) in 33/147 patients (22.4%) with EVF (14). These conflicting findings highlight the instability of the association, likely attributable to differences in sample size, baseline characteristics, and geographic regions. Shimonaga et al. reported a significantly lower rate of good prognosis in the EVF group compared to the non-EVF group (23 vs. 69%, *P* = 0.006) (15). This study included 35 patients, all cardioembolic, which limited both the reliability and generalizability of the results. Similarly, Ohta et al. observed a significantly lower rate of good prognosis (22.6 vs. 58.9%, *P* < 0.001) among 31/104 patients (29.8%) with EVF (11). However, the study included patients with MCA occlusion with postoperative reperfusion TIMI grades 0–3, a difference that likewise compromises the results in terms of reliability and generalizability. Nevertheless, both studies suggest that EVF may have prognostic relevance in patients with specific etiologies or specific vessels.

Cartmell et al. (8) reported that EVF correlates with poor prognosis, though to a lesser extent. The inclusion criteria for this study involved patients with a Thrombolysis in Cerebral Infarction (TICI) grades I–III. Patients with TICI grade I typically have poor reperfusion, which itself may be associated with a poor prognosis. This factor may have influenced the correlation between prognosis and EVF. Additionally, EVF was only one of many factors in prognostic outcomes in the study, and it was difficult to extract data specific to EVF. Therefore, it was not included in our current meta-analysis of 90-day functional independence.

Current evidence regarding the relationship between EVF and clinical prognosis remains inconsistent. Therefore, we conducted a thorough literature review, including six original studies with a total of 1,101 patients. The meta-analysis demonstrated that EVF may be associated with a lower likelihood of achieving 90-day functional independence (OR 0.42, 95% CI 0.18–0.99, *P* = 0.05). Accordingly, the intraoperative observation of EVF may warrant increased clinical vigilance, particularly with respect to hemorrhagic risk and peri-procedural management.

Unlike traditional predictors of reperfusion injury that are typically assessed postoperatively, EVF is a real-time intraoperative angiographic marker observed directly during thrombectomy. This unique temporal characteristic allows EVF to complement established prognostic factors by providing immediate feedback during the procedure. Furthermore, by synthesizing all available evidence, our study provides the first pooled quantitative assessment of the association between EVF, hemorrhagic complications, and 90-day functional independence, offering a more integrated understanding of its clinical significance beyond prior single-center observations.

On the other hand, Fritzsch et al. (22) observed in their study a significant attenuation of EVF 7–25 min after middle cerebral artery recanalization. A case report indicated that EVF was not detected in a patient who underwent DSA review 2 weeks later (23). Combined with the possible correlation between EVF and areas of postoperative hyperperfusion (15), it is hypothesized that EVF-associated postoperative hyperperfusion may similarly attenuate or be absent. This phenomenon may also occur in patients undergoing angioplasty. This suggests that if a significant EVF is observed during mechanical thrombolysis and angioplasty, a safer staged procedure could be considered, with vascular preconditioning followed by further balloon dilation or stenting on an elective basis after hemodynamic stabilization.

## Limitations

This meta-analysis has several limitations. First, all included studies were retrospective cohort studies, which may have introduced inherent biases and unmeasured confounding. Second, only six studies were available, limiting statistical power. Third, key baseline variables—such as NIHSS, ASPECTS, collateral status, and antithrombotic exposure—were incompletely or inconsistently reported, and four studies lacked any baseline comparison between EVF and non-EVF groups, increasing the risk of residual confounding. Fourth, substantial methodological variability existed across studies, including differences in EVF definitions, imaging acquisition protocols, reperfusion grading criteria, and postoperative assessment timelines, which may have contributed to the observed heterogeneity and limited the interpretability of pooled estimates. Therefore, the present findings should be considered exploratory. Large prospective multicenter studies with standardized EVF definitions, comprehensive baseline reporting, and multivariable adjustment are needed to better characterize the prognostic relevance of EVF.

## Conclusion

EVF appears to be associated with an increased incidence of postoperative ICH and SICH, as well as a lower likelihood of achieving 90-day functional independence. However, given the retrospective nature of the available studies, these findings should be interpreted with caution. EVF should therefore be regarded as a potential intraoperative angiographic marker rather than a proven independent predictor of prognosis. Further prospective, standardized studies are needed to determine whether EVF has independent prognostic significance.

## Data Availability

The dataset used in this meta-analysis is not publicly available, as it was generated based on aggregated data extracted from published studies. No individual participant data or raw data from the original studies were accessed or shared. Therefore, we do not have the rights to make these data publicly available. This limitation reflects the inherent constraints of secondary data analysis using published literature. All data sources are fully referenced in the manuscript, and any data inquiries should refer to the original published studies, further inquiries can be directed to the corresponding author.

## References

[1] Saini V Guada L Yavagal DR. Global epidemiology of stroke and access to acute ischemic stroke interventions. Neurology. (2021) 97(20 Suppl 2):S6–16. doi: 10.1212/WNL.0000000000012781 34785599

[2] Herpich F Rincon F. Management of acute ischemic stroke. Crit Care Med. (2020) 48:1654–63. doi: 10.1097/CCM.0000000000004597 32947473 PMC7540624

[3] Turc G Bhogal P Fischer U Khatri P Lobotesis K Mazighi M . European stroke organisation (ESO)—European society for minimally invasive neurological therapy (ESMINT) guidelines on mechanical thrombectomy in acute ischemic stroke. J Neurointerv Surg. (2023) 15:e8. doi: 10.1136/neurintsurg-2018-014569 30808653

[4] Nogueira RG Jadhav AP Haussen DC Bonafe A Budzik RF Bhuva P . Thrombectomy 6 to 24 hours after stroke with a mismatch between deficit and infarct. New England J Med. (2018) 378:11–21. doi: 10.1056/NEJMoa1706442 29129157

[5] Rodriguez-Calienes A Galecio-Castillo M Vivanco-Suarez J Mohamed GA Toth G Sarraj A . Endovascular thrombectomy beyond 24 hours from last known well: a systematic review with meta-analysis. J Neurointerv Surg. (2024) 16:670–6. doi: 10.1136/jnis-2023-020443 37355251

[6] Fuhrer H Forner L Pruellage P Weber S Beume LA Schacht H . Long-term outcome changes after mechanical thrombectomy for anterior circulation acute ischemic stroke. J Neurol. (2020) 267:1026–34. doi: 10.1007/s00415-019-09670-w 31834520

[7] Behme D Gondecki L Fiethen S Kowoll A Mpotsaris A Weber W. Complications of mechanical thrombectomy for acute ischemic stroke—a retrospective single-center study of 176 consecutive cases. Neuroradiology. (2014) 56:467–76. doi: 10.1007/s00234-014-1352-0 24668181

[8] Cartmell SCD Ball RL Kaimal R Telischak NA Marks MP Do HM . Early Cerebral vein after endovascular ischemic stroke treatment predicts symptomatic reperfusion hemorrhage. Stroke. (2018) 49:1741–6. doi: 10.1161/STROKEAHA.118.021402 29739912

[9] Page MJ McKenzie JE Bossuyt PM Boutron I Hoffmann TC Mulrow CD . The PRISMA 2020 statement: an updated guideline for reporting systematic reviews. Syst Rev. (2021) 10:89. doi: 10.1186/s13643-021-01626-4 33781348 PMC8008539

[10] Stang A. Critical evaluation of the Newcastle-Ottawa scale for the assessment of the quality of nonrandomized studies in meta-analyses. Euro J Epidemiol. (2010) 25:603–5. doi: 10.1007/s10654-010-9491-z 20652370

[11] Ohta H Nakano S Yokogami K Iseda T Yoneyama T Wakisaka S. Appearance of early venous filling during intra-arterial reperfusion therapy for acute middle cerebral artery occlusion—a predictive sign for hemorrhagic complications. Stroke. (2004) 35:893–8. doi: 10.1161/01.STR.0000119751.92640.7F 14976322

[12] Li Y Cao W Xu X Li T Chen Y Wang Y . Early venous filling after mechanical thrombectomy in acute ischemic stroke due to large vessel occlusion in anterior circulation. J Neurointerv Surg. (2024) 16:248–52. doi: 10.1136/jnis-2023-020336 37197935

[13] Janvier P Kerleroux B Turc G Pasi M Farhat W Bricout N . TAGE score for symptomatic intracranial hemorrhage prediction after successful endovascular treatment in acute ischemic stroke. Stroke. (2022) 53:2809–17. doi: 10.1161/STROKEAHA.121.038088 35698971

[14] Elands S Casimir P Bonnet T Mine B Lubicz B Sjøgård M . Early venous filling following thrombectomy: association with hemorrhagic transformation and functional outcome. Front Neurol. (2021) 12:649079. doi: 10.3389/fneur.2021.649079 33776899 PMC7987949

[15] Shimonaga K Matsushige T Takahashi H Hashimoto Y Mizoue T Ono C . Early venous filling after reperfusion therapy in acute ischemic stroke. J Stroke Cerebrovasc Dis. (2020) 29:104926. doi: 10.1016/j.jstrokecerebrovasdis.2020.104926 32689637

[16] Charbonnier G Bonnet L Biondi A Moulin T. Intracranial bleeding after reperfusion therapy in acute ischemic stroke. Front Neurol. (2020) 11:629920. doi: 10.3389/fneur.2020.629920 33633661 PMC7900408

[17] van Kranendonk KR Treurniet KM Boers AMM Berkhemer OA van den Berg LA Chalos V . Hemorrhagic transformation is associated with poor functional outcome in patients with acute ischemic stroke due to a large vessel occlusion. J NeuroInterv Surg. (2019) 11:464–8. doi: 10.1136/neurintsurg-2018-014141 30297537

[18] Khatri P Wechsler LR Broderick JP. Intracranial hemorrhage associated with revascularization therapies. Stroke. (2007) 38:431–40. doi: 10.1161/01.STR.0000254524.23708.c9 17234988

[19] Bolaños JP Almeida A. Roles of nitric oxide in brain hypoxia-ischemia. Biochim Biophys Acta. (1999) 1411:415–36. doi: 10.1016/S0005-2728(99)00030-4 10320673

[20] Khatri R McKinney AM Swenson B Janardhan V. Blood–brain barrier, reperfusion injury, and hemorrhagic transformation in acute ischemic stroke. Neurology. (2012) 79(13_supplement_1):S52–57. doi: 10.1212/WNL.0b013e3182697e70 23008413

[21] Castro P Azevedo E Serrador J Rocha I Sorond F. Hemorrhagic transformation and cerebral edema in acute ischemic stroke: link to cerebral autoregulation. J Neurol Sci. (2017) 372:256–61. doi: 10.1016/j.jns.2016.11.065 28017224 PMC5310259

[22] Fritzsch D Reiss-Zimmermann M Lobsien D Quäschling U Hoffmann KT. Arteriovenous shunts and capillary blush as an early sign of basal ganglia infarction after successful mechanical intra-arterial thrombectomy in ischaemic stroke. Eur Radiol. (2015) 25:3060–5. doi: 10.1007/s00330-015-3704-5 26115652

[23] Chen H Xu Z. A case of reversible changes in early venous filling phenomenon after emergency endovascular therapy. Chinese J Neurol. (2020) 53:115–8.

