# Supplementary material for: Relationship between early venous filling after thrombectomy and intracranial hemorrhage and prognosis in patients with acute ischemic stroke: a systematic review and meta-analysis
Source: Front Neurol. 2026 Jan 14;16:1647906. doi: 10.3389/fneur.2025.1647906 (PMC12848917; doi:10.3389/fneur.2025.1647906)
Supplement: Supplementary file 1 [file Supplementary_file_1.docx]

**Relationship Between Early Venous Filling After Thrombectomy and Intracranial Hemorrhage and Prognosis in Patients with Acute Ischemic Stroke: A Systematic Review and Meta-Analysis**

**Supplementary Table 1.** Search strategy for each database.

| Pubmed | | |
| --- | --- | --- |
| Step | Search query | Results |
| #1 | "Early Diagnosis"[Mesh] | 72466 |
| #2 | ((Diagnosis, Early[Title/Abstract]) OR (Early Detection of Disease[Title/Abstract])) OR (Disease Early Detection[Title/Abstract]) | 2835 |
| #3 | early[Title/Abstract] | 1971295 |
| #4 | (("Early Diagnosis"[Mesh]) OR (((Diagnosis, Early[Title/Abstract]) OR (Early Detection of Disease[Title/Abstract])) OR (Disease Early Detection[Title/Abstract]))) OR (early[Title/Abstract]) | 2007031 |
| #5 | venous filling[Title/Abstract] | 458 |
| #6 | ((("Early Diagnosis"[Mesh]) OR (((Diagnosis, Early[Title/Abstract]) OR (Early Detection of Disease[Title/Abstract])) OR (Disease Early Detection[Title/Abstract]))) OR (early[Title/Abstract])) AND (venous filling[Title/Abstract]) | 95 |
| #7 | Peri-ischemic[Title/Abstract] | 191 |
| #8 | (Peri-ischemic[Title/Abstract]) AND (((("Early Diagnosis"[Mesh]) OR (((Diagnosis, Early[Title/Abstract]) OR (Early Detection of Disease[Title/Abstract])) OR (Disease Early Detection[Title/Abstract]))) OR (early[Title/Abstract])) AND (venous filling[Title/Abstract])) | 1 |
| #9 | "Perfusion"[Mesh] | 67615 |
| #10 | ("Perfusion"[Mesh]) OR (perfusions[Title/Abstract]) | 69292 |
| #11 | (luxury[Title/Abstract]) AND (("Perfusion"[Mesh]) OR (perfusions[Title/Abstract])) | 6 |
| #12 | ((((("Early Diagnosis"[Mesh]) OR (((Diagnosis, Early[Title/Abstract]) OR (Early Detection of Disease[Title/Abstract])) OR (Disease Early Detection[Title/Abstract]))) OR (early[Title/Abstract])) AND (venous filling[Title/Abstract])) OR ((Peri-ischemic[Title/Abstract]) AND (((("Early Diagnosis"[Mesh]) OR (((Diagnosis, Early[Title/Abstract]) OR (Early Detection of Disease[Title/Abstract])) OR (Disease Early Detection[Title/Abstract]))) OR (early[Title/Abstract])) AND (venous filling[Title/Abstract])))) OR ((luxury[Title/Abstract]) AND (("Perfusion"[Mesh]) OR (perfusions[Title/Abstract]))) | 101 |
| #13 | "Ischemic Stroke"[Mesh] | 14890 |
| #14 | （Ischemic Strokes[Title/Abstract]） OR （Stroke, Ischemic[Title/Abstract]） OR （Ischaemic Stroke[Title/Abstract]） OR （Ischaemic Strokes[Title/Abstract]） OR （Stroke, Ischaemic[Title/Abstract]） OR （Acute Ischemic Stroke[Title/Abstract]） OR （Acute Ischemic Strokes[Title/Abstract]） OR （Ischemic Stroke, Acute[Title/Abstract]） OR （Stroke, Acute Ischemic[Title/Abstract]） OR （Cryptogenic Ischemic Strokes[Title/Abstract]） OR （Cryptogenic Ischemic Stroke[Title/Abstract]） OR （Ischemic Stroke, Cryptogenic[Title/Abstract]） OR （Stroke, Cryptogenic Ischemic[Title/Abstract]） OR （Cryptogenic Embolism Stroke[Title/Abstract]） OR （Cryptogenic Embolism Strokes[Title/Abstract]） OR （Embolism Stroke, Cryptogenic[Title/Abstract]） OR （Stroke, Cryptogenic Embolism[Title/Abstract]） OR （Cryptogenic Stroke[Title/Abstract]） OR （Cryptogenic Strokes[Title/Abstract]） OR （Stroke, Cryptogenic[Title/Abstract]） OR （Wake-up Stroke[Title/Abstract]） OR （Stroke, Wake-up[Title/Abstract]） OR （Wake up Stroke[Title/Abstract]） OR （Wake-up Strokes[Title/Abstract]） | 40563 |
| #15 | ("Ischemic Stroke"[Mesh]) OR (（Ischemic Strokes[Title/Abstract]） OR （Stroke, Ischemic[Title/Abstract]） OR （Ischaemic Stroke[Title/Abstract]） OR （Ischaemic Strokes[Title/Abstract]） OR （Stroke, Ischaemic[Title/Abstract]） OR （Acute Ischemic Stroke[Title/Abstract]） OR （Acute Ischemic Strokes[Title/Abstract]） OR （Ischemic Stroke, Acute[Title/Abstract]） OR （Stroke, Acute Ischemic[Title/Abstract]） OR （Cryptogenic Ischemic Strokes[Title/Abstract]） OR （Cryptogenic Ischemic Stroke[Title/Abstract]） OR （Ischemic Stroke, Cryptogenic[Title/Abstract]） OR （Stroke, Cryptogenic Ischemic[Title/Abstract]） OR （Cryptogenic Embolism Stroke[Title/Abstract]） OR （Cryptogenic Embolism Strokes[Title/Abstract]） OR （Embolism Stroke, Cryptogenic[Title/Abstract]） OR （Stroke, Cryptogenic Embolism[Title/Abstract]） OR （Cryptogenic Stroke[Title/Abstract]） OR （Cryptogenic Strokes[Title/Abstract]） OR （Stroke, Cryptogenic[Title/Abstract]） OR （Wake-up Stroke[Title/Abstract]） OR （Stroke, Wake-up[Title/Abstract]） OR （Wake up Stroke[Title/Abstract]） OR （Wake-up Strokes[Title/Abstract]）) | 47665 |
| #16 | (acute[Title/Abstract]) AND (("Ischemic Stroke"[Mesh]) OR (（Ischemic Strokes[Title/Abstract]） OR （Stroke, Ischemic[Title/Abstract]） OR （Ischaemic Stroke[Title/Abstract]） OR （Ischaemic Strokes[Title/Abstract]） OR （Stroke, Ischaemic[Title/Abstract]） OR （Acute Ischemic Stroke[Title/Abstract]） OR （Acute Ischemic Strokes[Title/Abstract]） OR （Ischemic Stroke, Acute[Title/Abstract]） OR （Stroke, Acute Ischemic[Title/Abstract]） OR （Cryptogenic Ischemic Strokes[Title/Abstract]） OR （Cryptogenic Ischemic Stroke[Title/Abstract]） OR （Ischemic Stroke, Cryptogenic[Title/Abstract]） OR （Stroke, Cryptogenic Ischemic[Title/Abstract]） OR （Cryptogenic Embolism Stroke[Title/Abstract]） OR （Cryptogenic Embolism Strokes[Title/Abstract]） OR （Embolism Stroke, Cryptogenic[Title/Abstract]） OR （Stroke, Cryptogenic Embolism[Title/Abstract]） OR （Cryptogenic Stroke[Title/Abstract]） OR （Cryptogenic Strokes[Title/Abstract]） OR （Stroke, Cryptogenic[Title/Abstract]） OR （Wake-up Stroke[Title/Abstract]） OR （Stroke, Wake-up[Title/Abstract]） OR （Wake up Stroke[Title/Abstract]） OR （Wake-up Strokes[Title/Abstract]）)) | 31250 |
| #17 | ((acute[Title/Abstract]) AND (("Ischemic Stroke"[Mesh]) OR (（Ischemic Strokes[Title/Abstract]） OR （Stroke, Ischemic[Title/Abstract]） OR （Ischaemic Stroke[Title/Abstract]） OR （Ischaemic Strokes[Title/Abstract]） OR （Stroke, Ischaemic[Title/Abstract]） OR （Acute Ischemic Stroke[Title/Abstract]） OR （Acute Ischemic Strokes[Title/Abstract]） OR （Ischemic Stroke, Acute[Title/Abstract]） OR （Stroke, Acute Ischemic[Title/Abstract]） OR （Cryptogenic Ischemic Strokes[Title/Abstract]） OR （Cryptogenic Ischemic Stroke[Title/Abstract]） OR （Ischemic Stroke, Cryptogenic[Title/Abstract]） OR （Stroke, Cryptogenic Ischemic[Title/Abstract]） OR （Cryptogenic Embolism Stroke[Title/Abstract]） OR （Cryptogenic Embolism Strokes[Title/Abstract]） OR （Embolism Stroke, Cryptogenic[Title/Abstract]） OR （Stroke, Cryptogenic Embolism[Title/Abstract]） OR （Cryptogenic Stroke[Title/Abstract]） OR （Cryptogenic Strokes[Title/Abstract]） OR （Stroke, Cryptogenic[Title/Abstract]） OR （Wake-up Stroke[Title/Abstract]） OR （Stroke, Wake-up[Title/Abstract]） OR （Wake up Stroke[Title/Abstract]） OR （Wake-up Strokes[Title/Abstract]）))) AND (((((("Early Diagnosis"[Mesh]) OR (((Diagnosis, Early[Title/Abstract]) OR (Early Detection of Disease[Title/Abstract])) OR (Disease Early Detection[Title/Abstract]))) OR (early[Title/Abstract])) AND (venous filling[Title/Abstract])) OR ((Peri-ischemic[Title/Abstract]) AND (((("Early Diagnosis"[Mesh]) OR (((Diagnosis, Early[Title/Abstract]) OR (Early Detection of Disease[Title/Abstract])) OR (Disease Early Detection[Title/Abstract]))) OR (early[Title/Abstract])) AND (venous filling[Title/Abstract])))) OR ((luxury[Title/Abstract]) AND (("Perfusion"[Mesh]) OR (perfusions[Title/Abstract])))) | 6 |

| Cochrane Library | | |
| --- | --- | --- |
| Step | Search query | Results |
| #1 | (early or Diagnosis, Early or Early Detection of Disease or Disease Early Detection):ti,ab,kw | 156952 |
| #2 | (venous filling):ti,ab,kw | 702 |
| #3 | #1 and #2 | 95 |
| #4 | (Peri-ischemic):ti,ab,kw | 1 |
| #5 | #4 and #3 | 0 |
| #6 | MeSH descriptor: [Perfusion] explode all trees | 1387 |
| #7 | (perfusion or perfusions):ti,ab,kw | 15783 |
| #8 | #6 or #7 | 16325 |
| #9 | (luxury):ti,ab,kw | 52 |
| #10 | #9 and #8 | 11 |
| #11 | #3 or #5 or #10 | 106 |
| #12 | MeSH descriptor: [Ischemic Stroke] explode all trees | 1618 |
| #13 | (Ischemic stroke or Ischemic Strokes or Stroke, Ischemic or Ischaemic Stroke or Ischaemic Strokes or Stroke, Ischaemic or Acute Ischemic Stroke or Acute Ischemic Strokes or Ischemic Stroke, Acute or Stroke, Acute Ischemic or Cryptogenic Ischemic Stroke or Cryptogenic Ischemic Strokes or Ischemic Stroke, Cryptogenic or Stroke, Cryptogenic Ischemic or Cryptogenic Embolism Stroke or Cryptogenic Embolism Strokes or Embolism Stroke, Cryptogenic or Stroke, Cryptogenic Embolism or Cryptogenic Stroke or Cryptogenic Strokes or Stroke, Cryptogenic or Wake-up Stroke or Stroke, Wake-up or Wake up Stroke or Wake-up Strokes):ti,ab,kw | 21042 |
| #14 | #12 or #13 | 21092 |
| #15 | (acute):ti,ab,kw | 180663 |
| #16 | #15 and #14 | 10358 |
| #17 | #16 and #11 | 2 |

| Web of science | | |
| --- | --- | --- |
| Step | Search query | Results |
| #1 | Diagnosis, Early or Early Detection of Disease or Disease Early Detection or early diagnosis or early (主题) | 5815775 |
| #2 | (#1) AND TS=(venous filling) | 1254 |
| #3 | (#2) AND TS=(Peri-ischemic) | 1 |
| #4 | (TS=(luxury)) AND TS=(perfusion or perfusions) | 304 |
| #5 | #4 OR #3 OR #2 | 1554 |
| #6 | (TS=(Ischemic stroke or Ischemic Strokes or Stroke, Ischemic or Ischaemic Stroke or Ischaemic Strokes or Stroke, Ischaemic or Acute Ischemic Stroke or Acute Ischemic Strokes or Ischemic Stroke, Acute or Stroke, Acute Ischemic or Cryptogenic Ischemic Stroke or Cryptogenic Ischemic Strokes or Ischemic Stroke, Cryptogenic or Stroke, Cryptogenic Ischemic or Cryptogenic Embolism Stroke or Cryptogenic Embolism Strokes or Embolism Stroke, Cryptogenic or Stroke, Cryptogenic Embolism or Cryptogenic Stroke or Cryptogenic Strokes or Stroke, Cryptogenic or Wake-up Stroke or Stroke, Wake-up or Wake up Stroke or Wake-up Strokes)) AND TS=(acute) | 94691 |
| #7 | #6 AND #5 | 53 |

| Embase | | |
| --- | --- | --- |
| Step | Search query | Results |
| #1 | 'early':ab,ti OR 'diagnosis, early':ab,ti OR 'early detection of disease':ab,ti OR 'disease early detection':ab,ti | 2,704,003 |
| #2 | 'venous filling' | 628 |
| #3 | #1 AND #2 | 146 |
| #4 | 'peri ischemic' | 249 |
| #5 | #3 AND #4 | 1 |
| #6 | 'perfusion'/exp OR 'perfusion' | 421,236 |
| #7 | 'perfusions' | 3936 |
| #8 | #6 OR #7 | 422,259 |
| #9 | 'luxury' | 2,165 |
| #10 | #8 AND #9 | 254 |
| #11 | #3 OR #5 OR #10 | 397 |
| #12 | 'ischemic stroke'/exp | 36,780 |
| #13 | 'ischemic stroke':ab,ti OR 'ischemic strokes':ab,ti OR 'stroke, ischemic':ab,ti OR 'ischaemic stroke':ab,ti OR 'ischaemic strokes':ab,ti OR 'stroke, ischaemic':ab,ti OR 'acute ischemic stroke':ab,ti OR 'acute ischemic strokes':ab,ti OR 'ischemic stroke, acute':ab,ti OR 'stroke, acute ischemic':ab,ti OR 'cryptogenic ischemic stroke':ab,ti OR 'cryptogenic ischemic strokes':ab,ti OR 'ischemic stroke, cryptogenic':ab,ti OR 'stroke, cryptogenic ischemic':ab,ti OR 'cryptogenic embolism stroke':ab,ti OR 'cryptogenic embolism strokes':ab,ti OR 'embolism stroke, cryptogenic':ab,ti OR 'stroke, cryptogenic embolism':ab,ti OR 'cryptogenic stroke':ab,ti OR 'cryptogenic strokes':ab,ti OR 'stroke, cryptogenic':ab,ti OR 'wake-up stroke':ab,ti OR 'stroke, wake-up':ab,ti OR 'wake up stroke':ab,ti | 138,527 |
| #14 | #12 OR #13 | 147,262 |
| #15 | 'acute' | 2,575,386 |
| #16 | #14 AND #15 | 72,646 |
| #17 | #11 AND #16 | 30 |

**Supplementary Table 2.** Clinical characteristics between EVF and Non-EVF groups

| Characteristic | Author | EVF | N-EVF | P value |
| --- | --- | --- | --- | --- |
| Male gender,n(%) | Li et al., 2023^12^ | 23（51.11） | 211（69.41） | 0.31 |
|  | Elands et al., 2021^14^ | 11（33.33） | 35（30.70） |  |
|  | Shimonaga et al., 2020^15^ | 10（45.45） | 5（38.46） |  |
|  | Ohta et al., 2004^11^ | 19（61.29） | 41（56.16） |  |
| HTN,n(%) | Li et al., 2023^12^ | 27（60.00） | 198（65.13） | 0.91 |
|  | Elands et al., 2021^14^ | 26（78.79） | 79（69.30） |  |
| DM,n(%) | Li et al., 2023^12^ | 9（20.00） | 82（26.97） | 0.22 |
|  | Elands et al., 2021^14^ | 4（12.12） | 20（17.54） |  |
| CAD,n(%) | Li et al., 2023^12^ | 18（40.00） | 68（22.37） | 0.19 |
|  | Elands et al., 2021^14^ | 10（30.30） | 33（28.95） |  |
| AF,n(%) | Li et al., 2023^12^ | 16（35.56） | 79（25.99） | 0.066 |
|  | Elands et al., 2021^14^ | 18（54.55） | 48（42.11） |  |
| Smoking,n(%) | Li et al., 2023^12^ | 14（31.11） | 124（40.79） | 0.03* |
|  | Elands et al., 2021^14^ | 6（18.18） | 43（37.72） |  |
| Stroke history,n(%) | Li et al., 2023^12^ | 9（20.00） | 55（18.09） | 0.81 |
|  | Elands et al., 2021^14^ | 4（12.12） | 14（12.28） |  |
| Antiplatelet use,n(%) | Li et al., 2023^12^ | 7（15.56） | 28（9.21） | 0.87 |
|  | Elands et al., 2021^14^ | 10（30.30） | 45（39.47） |  |
| Anticoagulation use,n(%) | Li et al., 2023^12^ | 11（24.44） | 62（20.39） | 0.96 |
|  | Elands et al., 2021^14^ | 5（15.15） | 25（21.93） |  |
| Intravenous thrombolysis,n(%) | Li et al., 2023^12^ | 20（44.44） | 114（37.50） | 0.22 |
|  | Elands et al., 2021^14^ | 10（30.30） | 26（22.81） |  |
| ICA Occlusion,n(%) | Li et al., 2023^12^ | 19（42.22） | 118（38.82） | 0.27 |
|  | Elands et al., 2021^14^ | 14（42.42） | 35（30.70） |  |
| MCA Occlusion,n(%) | Li et al., 2023^12^ | 26（57.78） | 184（60.53） | 0.3 |
|  | Elands et al., 2021^14^ | 19（57.58） | 79（69.30） |  |
| *： <0.05.  EVF, early venous filling; N-EVF, non- early venous filling; HTN, hypertension; DM, diabetes mellitus; CAD, coronary artery disease; AF, atrial fibrillation; ICA, internal carotid artery; MCA, middle cerebral artery. | | | | |

**Supplementary Table 3.** Quality assessment of the studies included (according to the Newcastle-Ottawa Scale [NOS] for cohort studies).

| Included studies | Selection | Comparability | Outcome | Total points |
| --- | --- | --- | --- | --- |
| Li et al., 2023^12^ | ★★★★ | ★★ | ★★★ | 9 |
| Cartmell et al., 2018^8^ | ★★★★ |  | ★★★ | 7 |
| Janvier et al., 2022^13^ | ★★★★ | ★ | ★★ | 7 |
| Elands et al., 2021^14^ | ★★★★ | ★★ | ★★★ | 9 |
| Shimonaga et al., 2020^15^ | ★★★★ |  | ★★★ | 7 |
| Ohta et al., 2004^11^ | ★★★★ |  | ★★★ | 7 |

**Supplementary Figure 1.** Adjusted funnel plot


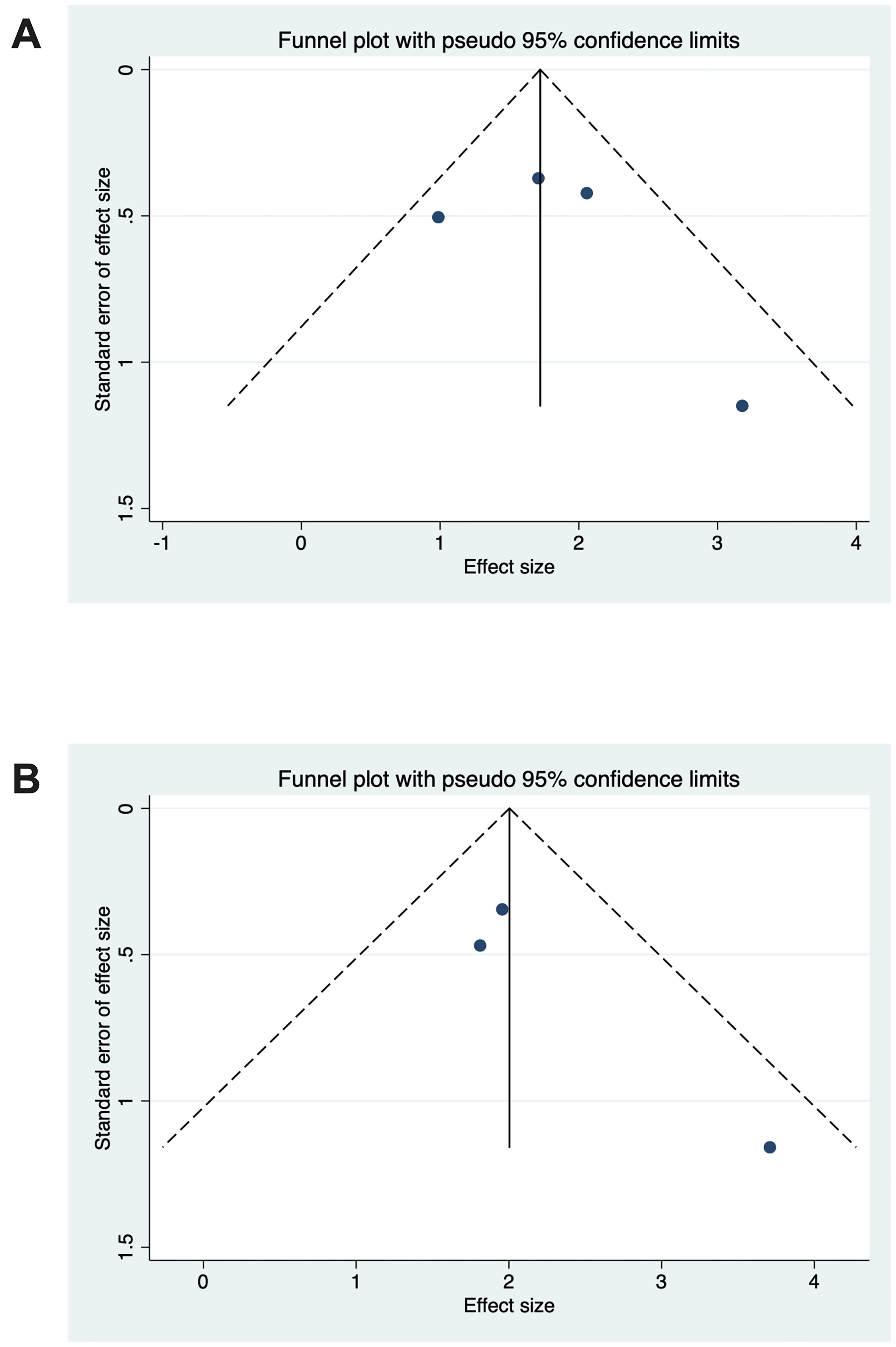


A： Symptomatic intracranial haemorrhage (SICH) outcomes.

B： Intracranial hemorrhage (ICH) outcome.
